# Efficacy of GAD-alum immunotherapy associated with *HLA-DR3-DQ2* in recently diagnosed type 1 diabetes

**DOI:** 10.1007/s00125-020-05227-z

**Published:** 2020-08-05

**Authors:** Ulf Hannelius, Craig A. Beam, Johnny Ludvigsson

**Affiliations:** 1 Diamyd Medical AB, Kungsgatan 29, 111 56 Stockholm, Sweden; 2 Department of Biomedical Sciences, Western Michigan University Homer Stryker M.D. School of Medicine, Kalamazoo, MI USA; 3 Crown Princess Victoria Children’s Hospital, Linköping, Sweden; 4 Division of Pediatrics, Department of Biomedical and Clinical Sciences (BKV), Medical Faculty, Linköping University, SE 58185 Linköping, Sweden

**Keywords:** Antigen-specific, Autoimmune diabetes, C-peptide, GAD, Glutamic acid decarboxylase, HLA, Immunotherapy, Type 1 diabetes, Vaccine

## Abstract

**Aims/hypothesis:**

The aim of this study was to determine if retention of C-peptide following immunotherapy using recombinant GAD65 conjugated to aluminium hydroxide (GAD-alum) is influenced by HLA risk haplotypes *DR3-DQ2* and *DR4-DQ8*.

**Methods:**

HLA-dependent treatment effect of GAD-alum therapy on C-peptide retention in individuals with recent-onset type 1 diabetes was evaluated using individual-level patient data from three placebo-controlled, randomised clinical trials using a mixed repeated measures model.

**Results:**

A significant and dose-dependent effect was observed in individuals positive for the genotypes that include *HLA-DR3-DQ2* but not *HLA-DR4-DQ8* and in the broader subgroup of individuals positive for all genotypes that include *HLA-DR3-DQ2* (i.e. including those also positive for *HLA-DR4-DQ8*). Higher doses (three or four injections) showed a treatment effect ratio of 1.596 (95% CI 1.132, 2.249; adjusted *p* = 0.0035) and 1.441 (95% CI 1.188, 1.749; adjusted *p* = 0.0007) vs placebo for the two respective HLA subgroups.

**Conclusions/interpretation:**

GAD65-specific immunotherapy has a significant effect on C-peptide retention in individuals with recent-onset type 1 diabetes who have the *DR3-DQ2* haplotype.

**Figure:**
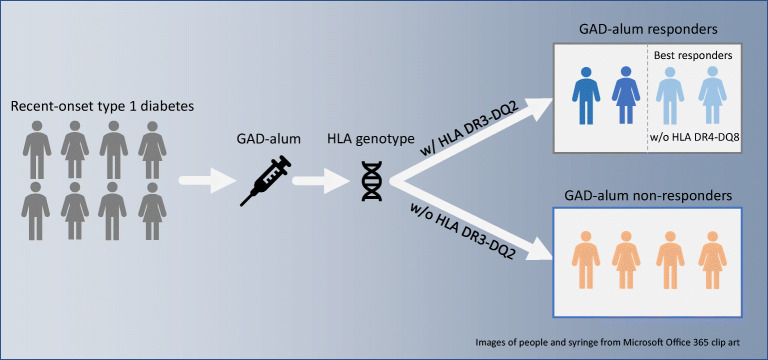
Graphical abstract

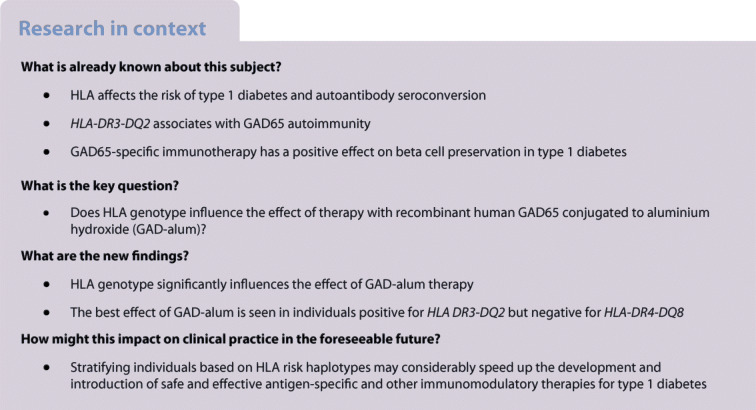



## Introduction

Recombinant human GAD65 conjugated to aluminium hydroxide (GAD-alum) is an antigen-specific immunotherapy intended to induce specific immunological tolerance to preserve the pancreatic beta cells that are targeted in type 1 diabetes by autoreactive cytotoxic T cells. The active ingredient, recombinant human GAD65, is a pancreatic beta cell protein, GAD65 being one of the most frequent autoantigens associated with type 1 diabetes. The effect of GAD-alum on preserving endogenous insulin production has been evaluated in several placebo-controlled, randomised trials in individuals recently diagnosed with type 1 diabetes, albeit with inconclusive results [1–4]. It is becoming increasingly clear that factors such as genetic background in the form of HLA genotype affect both the risk of diabetes affliction and the pathogenesis of the disease [5–13]. It is conceivable that HLA may also influence the effect of antigen-specific immunotherapies like GAD-alum and while this possibility has been considered incidentally in prior clinical trials [2, 3], this hypothesis has not been extensively evaluated.

The aim of this study was to estimate, using individual-level patient data from previous randomised, placebo-controlled trials, whether the efficacy of GAD-specific immunotherapy depends on the presence of the GAD and insulin antibody-associated HLA haplotypes *DR3-DQ2* and *DR4-DQ8*.

## Methods

We combined individual-level data from three published randomised, controlled clinical trials [2–4], clinical trial identifiers NCT00435981, NCT00529399 and NCT00723411, that evaluated subcutaneous GAD-alum therapy (compared with alum) in GAD autoantibody-positive individuals with recent-onset type 1 diabetes.

The similarities and differences between the clinical studies have been described in detail elsewhere [1]. Table 1 summarises the number of participants eligible for the analysis, treatment schedules and HLA genotype distribution. Briefly, all trials evaluated treatment with two injections of 20 μg GAD-alum or alum only (placebo). Two trials [2, 3] also evaluated three or four injections of 20 μg GAD-alum. GAD-alum or placebo was administered at days 1, 30, 90 and 270, respectively. For the analysis, the treatment was coded as placebo, low dose (two injections) and high dose (three or four injections).

**Table 1:** Summary of data, HLA genotype distribution and treatment regimens as evaluated in the analysis

	Ludvigsson et al, 2008 (NCT00435981) [4]	Wherrett et al, 2011 (NCT00529399) [3]	Ludvigsson et al, 2012 (NCT00723411) [2]
Participants eligible for analysis, *n*	69	139	313
All participants	70	145	334
With baseline and at least one post-baseline value missing	1	4	7
With missing HLA information	0	2	14
HLA distribution, *n* (%)			
*HLA-DR3-DQ2*	34 (49%)	71 (50%)	161 (50%)
*HLA-DR3-DQ2*/not *HLA-DR4-DQ8*	17 (24%)	36 (25%)	74 (23%)
Treatment schedule^a^			
Low dose	Two doses of 20 μg GAD-alum (days 1 and 30)	Two doses of 20 μg GAD-alum (days 1 and 30), one dose of alum (day 90)	Two doses of 20 μg GAD-alum (days 1 and 30), two doses of alum (days 90 and 180)
High dose	-	Three doses of 20 μg GAD-alum (days 1, 30 and 90)	Four doses of 20 μg GAD-alum (days 1, 30, 90 and 180)
Placebo	Two doses of alum (days 1 and 30)	Three doses of alum (days 1, 30 and 90)	Four doses of alum (days 1, 30, 90 and 180)

^a^For Wherrett et al 2011 the treatment schedule was defined as baseline, week 4 and week 12, while Ludvigsson 2008 and 2012 defined the schedule based on days from baseline (1, 30, 90 and 180). For consistency, days from baseline are used in the table. For a detailed description on the similarities and differences between the clinical trials, please see [1]

A total of 521 participants (of 549) were included in the final analysis. Patients that did not have a baseline and at least one post-baseline C-peptide value (*n* = 12 out of 549) were excluded as well as individuals with missing HLA information (*n* = 16 out of 537 remaining). The HLA subgroup term was coded as either presence or absence of genotypes that include *HLA-DR3-DQ2* and, in a second model, as either presence or absence of genotypes that include *HLA-DR3-DQ2* but not *HLA-DR4-DQ8*.

The treatment effect on C-peptide retention (log_*e*_ of the ratio of C-peptide AUC at 15 months/C-peptide AUC at baseline) was estimated using the restricted maximum likelihood approach in a mixed repeated measures model. All measurements from baseline to the primary endpoint readout (12 months for NCT00529399, 15 months for NCT00435981 and NCT00723411) were used. The model was adjusted for the fixed effects of baseline C-peptide, study, treatment, HLA subgroup, visit, country, sex and age, as well as the interaction of baseline C-peptide by visit and treatment by HLA subgroup by visit. Baseline value, age and visit were treated as continuous variables. Study, treatment, HLA subgroup, country and sex were treated as categorical variables. Patient identification number and country were included as categorical random effects to yield a variance components structure. The treatment effect ratios at 15 months were based on least square means with adjusted *p* values using the Bonferroni–Šidák correction reported along with two-sided 95% confidence intervals. A *p* value of <0.05 was considered significant. Statistical analysis was performed using SAS version 9.4 (SAS Institute, Cary, NC, USA).

## Results

**Fig. 1:**
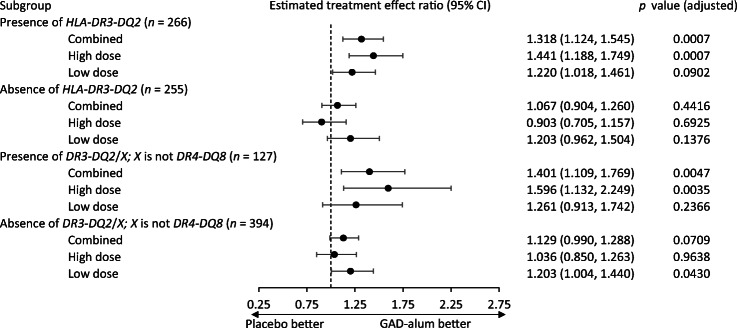
Estimated treatment effect ratio (C-peptide retention; active vs placebo) at 15 months post baseline in patients with or without genotypes that include *HLA-DR3-DQ2* (*n* = 266 and 255), or *HLA-DR3-DQ2* but not *HLA-DR4-DQ8* (*n* = 127 and *n* = 394). Adjusted *p* values using the Bonferroni- Šidák correction are reported along with two-sided 95% CIs

A significant interaction was observed in the main statistical model between treatment, C-peptide retention (log_*e*_ of the C-peptide AUC ratio 15 months/baseline), and the *HLA-DR3-DQ2* (*p* = 0.02) or *DR3-DQ2*/not *DR4-DQ8* genotype groups (*p* = 0.03), indicating that the treatment effect of GAD-alum differs depending on the patient’s HLA haplotype. Post hoc tests showed that the estimated treatment effect ratio (Fig. 1) of GAD-alum compared with placebo at 15 months from baseline was 1.318 (95% CI 1.124, 1.545; adjusted *p* = 0.0007) in individuals positive for *DR3-DQ2* (*n* = 266) genotype and 1.401 (95% CI 1.109, 1.769; adjusted *p* = 0.0047) in individuals positive for the *DR3-DQ2*/ not *DR4-DQ8* (*n* = 127) genotypes. A higher dose (three or four injections of GAD-alum) showed a treatment effect ratio of 1.441 (95% CI 1.188, 1.749; adjusted *p* = 0.0007) and 1.596 (95% CI 1.132, 2.249; adjusted *p* = 0.0035) vs placebo for the two HLA subgroups. No significant effect after adjusting for multiple testing was seen in these subgroups following a lower dose (two injections).

In individuals negative for the *DR3-DQ2*/not *DR4-DQ8* genotypes no significant effect was seen for any dose or for the combination of doses. For individuals negative for *DR3-DQ2* genotypes, a treatment effect ratio of 1.203 (95% CI 1.004, 1.440; adjusted *p* = 0.043) for lower dose was seen. No significance was seen for higher dose or for the combined dose regimen.

## Discussion

This study demonstrates that the efficacy of GAD-alum immunotherapy may depend on specific HLA risk haplotypes. The concept that HLA molecules play an important role in guiding antigen-specific autoimmunity in type 1 diabetes is not new as previous studies demonstrate that the first appearance of either GAD or insulin autoantibodies was associated with *HLA-DR3-DQ2* and *DR4-DQ8*, respectively [7]. However, this is the first report that we are aware of identifying the ability of specific HLA types to determine the efficacy of GAD-based therapeutics.

Specifically, based on this analysis where we combine individual patient-level data from three previous placebo-controlled clinical trials, we show that GAD-alum immunotherapy has a significant and dose-dependent effect on C-peptide retention in individuals positive for genotypes that include the *HLA-DR3-DQ2* haplotype. We further show that the effect is most pronounced in individuals who are simultaneously negative for genotypes that include the *HLA-DR4-DQ8* haplotype. Interestingly, recent research highlights the existence of different phenotypes of type 1 diabetes based on autoantibody seroconversion that, in turn, are linked to HLA risk haplotypes [7, 9, 10]. More specifically and as noted above, as *HLA-DR3-DQ2* has been associated with seroconversion to GAD65 autoantibodies and *DR4-DQ8* with seroconversion to insulin autoantibodies [7, 10], our results suggest that the best efficacy of antigen-specific immunotherapy may be achieved when targeting individuals that show a specific HLA type that is linked to the tolerising antigen.

These findings are in alignment with recent discussions highlighting the importance of considering disease heterogeneity in type 1 diabetes when evaluating therapeutic strategies [10–13]. Importantly, HLA genotyping is a straightforward and clinically feasible strategy to prospectively identify individuals with type 1 diabetes that have a higher likelihood of responding to the GAD-alum treatment.

Going forward, HLA information will be an integral part of any type 1 diabetes trial with GAD-alum. The current retrospective analyses involve comprehensive data from three randomised controlled trials conducted both in Europe and in the USA, with slightly different inclusion criteria. As these analyses were performed post hoc, it will be important to prospectively verify the findings presented here with regards to the interaction of HLA and GAD-alum therapy in ongoing trials. It will also be important to understand how HLA affects the immunological response to the therapy. Interestingly, preliminary findings (U. Hannelius, M. Danelljan, unpublished results) indicate that HLA does affect the GAD antibody response. There is also a clear rationale to retrospectively analyse the interaction between HLA and treatment in other antigen-specific trials, especially where insulin has been used as the tolerising antigen given the association between insulin autoimmunity and *HLA-DR4-DQ8*.

## Data Availability

The data that support the findings of this study are available on reasonable request from the corresponding author (UH).

## Abbreviation

GAD-alum: Recombinant human GAD65 conjugated to aluminium hydroxide
rhGAD65: Recombinant human GAD65
